# Faster waning of immunity to influenza vaccine strains reintroduced into subsequent seasons: A human cohort study

**DOI:** 10.1016/j.vaccine.2026.129258

**Published:** 2026-10-27

**Authors:** Cong Cheng, Xianyan Chen, Yang Ge, Meng-Hsuan Sung, Jiahui Zuo, Michael A. Carlock, Hannah B. Hanley, Ted M. Ross, Andreas Handel, Ye Shen

**Affiliations:** a Department of Statistics, Franklin College of Art and Science, University of Georgia, Athens, GA, United States; b Department of Epidemiology and Biostatistics, College of Public Health, University of Georgia, Athens, GA, United States; c Center for Vaccines and Immunology, University of Georgia, Athens, GA, United States; d Department of Infectious Diseases, University of Georgia, Athens, GA, United States; e Florida Research and Innovation Center, Cleveland Clinic, Port Saint Lucie, Florida, United States; f Department of Infection Biology, Lerner Research Institute, Cleveland Clinic, Cleveland, OH, United States; g Center for the Ecology of Infectious Diseases, University of Georgia, Athens, GA, United States of America

**Keywords:** Influenza vaccine, Hemagglutination inhibition assay, Antibody waning, Repeat vaccination, Multiyear cohort

## Abstract

**Background:**

Seasonal influenza vaccines are updated annually, and some seasons reuse strains from prior formulations. Although repeated vaccination has been associated with lower-than-expected post-vaccination titers, whether it alters immune durability remains unclear. We compared strain-specific waning rates across seasons in which vaccine formulations shared one or more strains.

**Methods:**

We analyzed hemagglutination inhibition (HAI) titers from a longitudinal vaccine cohort (2016–2023). For strains reused across seasons, we compared waning rate from season *i* to *i* + *1* (initial waning period) with waning from *i* + *1* to *i* + *2* (second waning period). Mixed-effects models estimated differences in strain-specific waning between periods, adjusting for age, BMI, previous vaccination in season *i − 1*, and other covariates.

**Results:**

Seven vaccine strains were reused across seasons (768 strain-specific measurements from 275 participants). The B lineage strain Phu/13 appeared in all seasons. Across repeatedly used subtype A and lineage B viruses, antibody levels waned significantly faster after subsequent vaccination. Among adults, the average increase in waning rate during the second period was 0.011 (H1N1), 0.108 (H3N2), 0.056 (Yamagata), and 0.049 (Victoria) log2 titer per month. Age modified these relationships, with slower waning in older adults for H1N1 and in teenagers for Yamagata. Waning also varied by strains, with BR08 waning faster than WA19. Higher pre-vaccination titers were associated with slower waning for H1N1 and H3N2, whereas larger post-vaccination boosts predicted faster waning for all virus strains.

**Conclusions:**

We found that the waning rates of certain vaccine strains may differ between initial and subsequent waning periods. Further investigation is warranted to optimize annual influenza vaccine composition updates.

## 1. Introduction

Influenza continues to be a major public health concern in the United States, contributing significantly to hospitalization and mortality each year [1]. Annual vaccination is recognized as the most effective strategy for preventing influenza infection and its complications, and is routinely recommended for nearly the entire population, especially individuals at high risk [2]. Influenza vaccine strain selection often involves reusing one or more strains from the previous season [3]. Immunological evidence indicates that primary and secondary exposures to an antigen elicit mechanistically distinct immune responses, particularly in the activation of naive versus memory B-cells [4]. However, it remains unclear whether reintroducing the same strain in subsequent seasonal influenza vaccines leads to same human immune responses.

Several studies found that a history of prior vaccination was associated with blunted vaccine effectiveness [5–10]. Concerns about the negative effects of repeated annual influenza vaccination were documented as early as the 1970s, when some studies found that prior vaccination was associated with infection risks comparable to, or greater than, those in unvaccinated individuals [11–13]. This negative effectiveness seems inconsistent across studies and seasons [14]. An eight-season human cohort study suggested that vaccine effectiveness against H3N2 was significantly higher among vaccinated individuals without prior vaccination history, whereas differences were smaller for the B strain [15]. A recent meta-analysis reported a 9% reduction in effectiveness for individuals vaccinated in two consecutive seasons compared to the first-time recipients [16].

For vaccine immunogenicity, the hemagglutination inhibition (HAI) titers were higher or similar among previously vaccinated individuals and dependent on the vaccine strain component [17]. However, while prior year vaccine recipients seemed to have better baseline antibody level in the current year, their absolute immune response increase associated with the current vaccine was relatively lower [18]. Our research team has previously calculated a HAI composite score to measure the strength of seasonal vaccine immunogenicity, and observed that the magnitude of response declined in the second season, with variations by age, gender, and BMI [19–21]. In general, and with some uncertainty [17,22], repeated vaccination was associated with non-superior short-term immune responses.

Much of the existing research on repeated vaccination relies on broad vaccination histories rather than detailed records of exposure to specific, repeatedly administered vaccine strains. Similarly, while some immunological studies have suggested a shortened antibody half-life following repeated vaccination [23,24], there is limited epidemiological evidence on whether the specific practice of reusing vaccine strains in consecutive seasons alters the rate of waning.

As the seasonal influenza vaccine is the only vaccine recommended annually, a thorough understanding of the impact of reusing the same vaccine strain is critical for future policy. In this study, we used seven years of data from a longitudinal human vaccine cohort to measure strain-specific HAI antibody waning for vaccine components reused in consecutive seasons. We found that HAI antibodies against a reused strain waned more rapidly in its subsequent season of use, and that the waning rate was modified by participant age.

## 2. Methods

We analyzed serological data from the UGAFluVac cohort [20,25,26] to compare the waning rate of an influenza strain that was included in the vaccine for more than one season.

### 2.1. Study design

We analyzed serological data collected in the UGAFluVac cohort from 2016/17 to 2022/23 influenza seasons [20,25,26]. There were seven waning periods among these seasons. Eligible participants received the Fluzone^™^ (Sanofi Pasteur, Swiftwater, PA, U.S.) split-inactivated influenza vaccine (IIV) each season and provided serum samples pre-vaccination (Day 0) and post-vaccination (Day 21/28). All the vaccination is standard dose. To investigate the HAI antibody titer waning, we filtered participants to have paired samples from two consecutive seasons. More specifically, from season *i*, we extract each participant’s post-vaccination HAI tier against the reused vaccine strain in season *i* and the pre-vaccination titer in season *i* + 1, as well as post-vaccination titer in season *i* + 1 and pre-vaccination titer in season *i* + 2. In this framework, the initial waning period represents waning after the first observed vaccination with a given strain, whereas the second waning period represents waning after the subsequent vaccination with the same strain in the next season. Therefore, this comparison reflects repeated exposure to the same vaccine strain across consecutive seasons, rather than a direct contrast between individuals whose previous-season vaccine contained an identical strain and those whose previous-season vaccine contained an antigenically distinct strain. The initial waning-period group may include participants who received an antigenically distinct strain in the previous season as well as participants who were not vaccinated in the previous season; thus, prior vaccination history in this group is not expected to be 100%.

During the study period, the quadrivalent influenza vaccine contained two Influenza A subtypes (H1N1,H3N2) and two Influenza B lineages (Yamagata, Victoria). The full list of vaccine strains in these seasons is available in Table 1. There were seven vaccine strains, including MI/15 (H1N1), Vic/19 (H1N1), HK/14 (H3N2), Phu/13 (Yamagata), BR/08 (Victoria), CO/17 (Victoria), and WA/19 (Victoria), reused in more than one influenza season. For the Yamagata lineage, because the 2016–2017 season was not the first season in which the Phu/13 strain was used, we restricted the analysis to participants whose first documented Phu/13 vaccination occurred during the study period.

#### Waning rate.

To compare the waning of each specific strain in the UGAFluVac cohort, we define the waning rate as the rate of antibody decay from *t*_1_ (the post-vaccination titer time in season *i*) to *t*_2_ (the pre-vaccination titer time in season *i* + *1*). Raw HAI titers were log2-transformed prior to calculation:

$$\text{Waning Rate}=\frac{{\text{log}}_{2}(HAI_{t1})-{\text{log}}_{2}(HAI_{t2})}{t_{2}-t_{1}}$$

where *HAI*_*t*1_ and *HAI*_*t*2_ are the HAI titers at time points *t*_1_ and *t*_2_ (*t*_2_ > *t*_1_), respectively, and *t*_2_ – *t*_1_ represents the number of months between the two time points. The unit for waning rate is log2 titer per month. Higher waning rates indicate faster antibody decay. The waning period is defined as the time interval from the post-vaccination month in the current influenza season to the pre-vaccination month in the subsequent influenza season.

We expected the waning rates to be greater than 0, but due to various factors, including natural infection or measurement error, a few individuals had a pre-vaccination HAI titer in season *i* + *1* higher than their post-vaccination HAI titer in season *i, resulting* a negative waning rate. To account for potential biases, we (1) excluded negative waning rates from the primary analysis and (2) conducted a sensitivity analysis in which negative waning rates were reset to 0.

#### Retention ratio.

To facilitate interpretation and comparison across strains, we additionally summarize antibody decay using a 12-month retention ratio. It quantifies the proportion of antibody titer retained after one year relative to the initial post-vaccination level:

$$\text{Retention Ratio}=\frac{HAI_{12}}{HAI_{0}}=2^{-12\times \text{Waning Rate}}.$$

A retention ratio closer to 1 indicates slower antibody decay and better long-term persistence, whereas smaller values indicate faster waning over time.

#### Covariates.

Demographic covariates included age, BMI, sex, and vaccination history. Age was categorized as teenagers (under 18 years), adults (18–64 years), and the elderly (65 years and older). Vaccination history indicated whether a participant had been vaccinated one season before.

We also incorporated two serological covariates: the pre-vaccination titer and the boost status in season *i*. The pre-vaccination titer measured the antibody levels before the vaccination. The boost status evaluates whether the post-vaccination HAI titers (Day 21/28) was at least one dilution higher than the pre-vaccination titers (Day 0) within the same influenza season.

### 2.2. Statistical analysis

Our outcome was the strain-specific waning rate, and our main factor of interest was the strain-specific waning period (first waning period after the first vaccination with a given strain, second waning period after the second vaccination with the same strain, and so on). Additionally, we consider all available waning periods for Phu/13. In the model, waning period was represented by *dose* variables. We used linear mixed-effects models with restricted maximum likelihood (REML) estimation to compare the strain-specific waning rate across consecutive waning periods. These models can account for correlated observations within individuals. Covariates in the models include age, BMI, vaccination history (Yes/No), boost status (Yes/No), pre-vaccination titer.

We fit separate models for each subtype and lineage of type A and type B influenza virus. More specifically, model 1 included MI/15 and Vic/19; model 2 included HK/14; model 3 included Phu/13; and model 4 included BR/08, CO/17, and WA/19. The Phu/13 vaccine strain was used in all seasons. To assess if the waning rate was modified by age, we included an interaction (e.g. Dose × Age) and evaluated its contribution by comparing model fit statistics.

All statistical analyses were performed using R software version 4.3.2 [27]. The lmerTest package [28] was used to fit the mixed effect models and provide *p*-values for fixed effects. The study data are available from the corresponding author upon reasonable request.

### 2.3. Ethics statement

This study is a secondary analysis of de-identified data. Participants were recruited at the UGA Clinical Trials Research Unit (CTRU) and the UGA Center for Vaccines and Immunology in Athens, GA, U.S. All participants enrolled provided written informed consent. The Institutional Review Board of the University of Georgia reviewed and approved the study procedures, informed consent, and data collection document. [20,25,26]

## 3. Results

### 3.1. Cohort characteristics

Table 2 summarizes the characteristics of participants exposed to reintroduced vaccine strains and the strain-specific HAI antibody titer measurements used for modeling. From the 2016/17 to 2022/23 influenza seasons, 275 participants were exposed at least once to a reintroduced vaccine strain. From these participants, 1276 strain-specific HAI antibody titer measurements were used for modeling. Adults constituted the majority of participants (ranging from 56.8% to 89.8%), followed by teenagers (0% - 39.7%) and older adults (0% - 11.1%). Females comprised more than half of the participants. Most individuals (over 60%) reported a history of prior influenza vaccination before receiving the specific vaccine strains examined in this study.

### 3.2. HAI titer dynamics

Fig. 1 illustrates the magnitudes and temporal patterns of inter-season HAI immune dynamics by age group for each vaccine strain. Arrows show the waning (a series of waning periods) in HAI titers from a post-vaccination time point (day 21/28) to the pre-vaccination time point (Day 0 in the next influenza season). Overall, antibody waning in later periods was faster than in the first phase in HK/14, Vic/19 and WA/19, indicating accelerated antibody decay upon repeated exposure. In contrast, for MI/15 and BR/08, the elderly showed a slower titer decline in the later phases, reversing the pattern observed for the other strains. Age-stratified patterns further revealed that teenagers experienced higher HAI titers and a generally slower waning, whereas elderly participants started with lower HAI titers and showed more rapid titer loss in later phases, particularly with the CO/17 and WA/19 vaccine strains. The distributions of pre-vaccination HAI titers vaccination phase I and II are presented in Supplementary Fig. S1.

Strain-specific waning rates comparison between two subsequent influenza seasons.

Participants received the HK/14, MI/15, Vic/19, BR/08, CO/17, and WA/19 vaccine strains twice across two consecutive seasons. We built four mixed effects models, one for each influenza subtype and lineage. Table 3 shows the models evaluating the association between strain-specific waning rate and repeated vaccination (first vs. second dose, corresponding to initial and second waning periods), adjusting for demographic and host factors. For adults, the second-dose administration was significantly associated with faster waning for H3N2 subtype (0.108 log titer per month, *p* < 0.001), Yamagata (0.056, *p* < 0.001), and Victoria (0.049, *p* < 0.001) lineage. Correspondingly, the 12-months retention ratios in the second dose were reduced to approximately 0.41, 0.63, and 0.70 times those in the first dose, respectively. For H1N1 and Yamagata, significant interaction effects between age group and dose administration were identified.

Prior vaccination history was significantly associated with slower waning in Yamagata lineage (−0.049 log titer per month, *p* < 0.001). Higher pre-vaccination titers were associated with slower waning in H1N1 (−0.011, *p* < 0.01) and H3N2 (−0.030, *p* < 0.001), but not in Yamagata or Victoria. Positive boost was consistently associated with faster waning across all strains (coefficients 0.048–0.075; p from <0.05 to <0.001).

In the above main analysis, negative strain-specific waning was dropped. In the sensitivity analysis, when these negative rates were reset to zero, we obtained similar results (Supplementary Tables S1). In addition, we conducted a sensitivity analysis excluding individuals without prior vaccination history (Supplementary Table S2). To address the concern regarding model specification across subtypes, we further conducted a sensitivity analysis using a single mixed-effects model with subtype included as a random effect (Supplementary Table S3). To account for the potential ceiling effect of pre-vaccination antibody levels on vaccine-induced boosting, we further conducted a sensitivity analysis using pre-vac adjusted boost status, which yielded results consistent with the primary analysis (Supplementary Table S4).

#### Strain-specific waning rates comparison across seven vaccinations.

In our study period, the Phu/13 vaccine strain was used in seven influenza seasons. As fewer participants receiving more than five consecutive vaccinations, we combined data related to fifth and subsequent vaccinations into fifth or more groups. Similarly, we found faster waning after the second dose (0.067 log titer per month, *p* < 0.001 in adults). The teenager*second-dose term was significant (−0.079, *p* < 0.001), indicating slower waning after the second dose in younger participants compared to adults. Strain-specific waning rates after the third, fourth, and fifth or more doses were not significantly different from the first dose. These doses do not interact with age groups (Table 4).

## 4. Discussions

This study discovered distinct antibody-waning patterns following reintroduction of the same vaccine strain in subsequent influenza seasons. We found that the second waning period was associated with significantly faster waning rates than the initial waning period, with this effect modified by age, a relationship that held for all strains but showed an exception in H1N1. Additionally, waning rates were associated with prior vaccination history, pre-vaccination titer, and post-vaccination boost.

Based on a linear projection from the estimated waning rates, the projected time for post-vaccination HAI titers to decline to the conventional seroprotection threshold of 40 was shorter in the second waning period than in the first waning period by approximately 2.2 months for H1N1, 4.0 months for H3N2, 3.7 months for Yamagata, and 2.6 months for Victoria. This aligns with another study finding that the antibody half-life shortens with successive seasonal influenza vaccinations for the same influenza strain (e.g., from 32 months after the first dose to 9 months after the seventh dose) [24], underlining the need for follow-up strategies. A possible biological explanation for the accelerated decline in vaccine-induced immunity following repeated vaccination is that the initial vaccination may trigger a robust antibody response with broad cross-reactivity to multiple epitopes on the viral hemagglutinin protein [29]. In contrast, subsequent vaccinations may elicit a more limited but highly specific antibody response, which, while more effective at neutralizing the virus, may wane more rapidly over time [24]. Notably, in Phu/13 we found that only the second dose exhibited this pronounced effect, while the waning observed in subsequent doses was not statistically different than the initial waning period. However, our sample size was likely insufficient to secure the needed statistical power to assess waning beyond two seasons, highlighting the need for future research.

We also found that the waning patterns were significantly different across vaccine strains. Even within the same influenza subtype or lineage, different strains could exhibit distinct immunological behaviors over time. This variation was evident across our models. Notably, for H3N2, higher pre-vaccination titers were associated with slower waning, whereas subsequent-season vaccination was independently associated with faster waning after adjustment for pre-vaccination titer and other covariates. Thus, the higher pre-vaccination titers observed in the second phase would favor slower rather than faster waning and therefore do not explain the observed faster waning. Previous studies have largely examined influenza vaccine effectiveness at the seasonal level, focusing on how overall antibody wanes within a season, rather than assessing immune responses or effectiveness associated with specific vaccine strains [30,31]. A pooled analysis spanning five recent influenza seasons in US revealed a moderate reduction in vaccine effectiveness against influenza A(H3N2), a slight reduction against influenza B, and no discernible reduction against influenza A(H1N1) pdm09 [31].

Dose×Age interaction further suggested that the effect of a second dose on antibody waning varies across age groups. Specifically, compared to adults, elderly individuals experienced slower HAI titer waning after the second dose for H1N1 vaccine strains, while teenagers exhibited slower waning after the second dose for the B/Yamagata vaccine strain. These subtype- and lineage-specific age-interaction patterns could reflect differences in immune system status, and accumulated immune history across birth cohorts. However, the biological mechanisms underlying these differences could not be directly evaluated in the current study, and some age-by-strain subgroups were small. Therefore, these interaction findings should be interpreted cautiously and confirmed in larger cohorts.

We further observed that participants having a prior vaccination history experienced slower antibody decay, whereas those who mounted a strong post-vaccination response tended to exhibit more rapid subsequent waning. This is consistent with the findings of [32], which reported that individuals with higher post-vaccination antibody titers tend to experience faster short-term waning. One study reported faster waning among repeatedly vaccinated individuals, but only based on analyses of a single influenza strain (A(H1N1)pdm09) [24].

Several limitations of the current study should be acknowledged. First, most vaccine strains were only reintroduced twice, so we may not have sufficient statistical powers to study the repetition effect. Second, while Day 21/28 HAI titers are typically used to represent peak immunity, our waning rate may not accurately capture the whole waning trajectory due to individual variation of peak time. Third, our analysis focused exclusively on humoral immune responses, without accounting for cell-mediated immunity, which may also play an important role. In addition, we did not have complete vaccination history information for each participant. As the Phu/13 strain was first introduced during the 2015/16 influenza season, it is possible that some participants had prior exposure. Future studies would benefit from longer follow-up periods and larger sample sizes to better assess prior exposure and its effects.

In conclusion, we found that repeated exposure to the same vaccine strain may be associated with faster HAI titer waning over the subsequent influenza season. This finding highlights the need to account for potentially accelerated waning of immunity in annual vaccine composition decisions, particularly when a specific strain is under consideration for reintroduction. Future mechanistic studies are warranted, particularly to clarify the half-life of antibody-secreting cells and their contribution to antibody persistence after repeated vaccination.

## Supplementary Material

Appendix A Supplementary data

## Figures and Tables

**Fig. 1.**
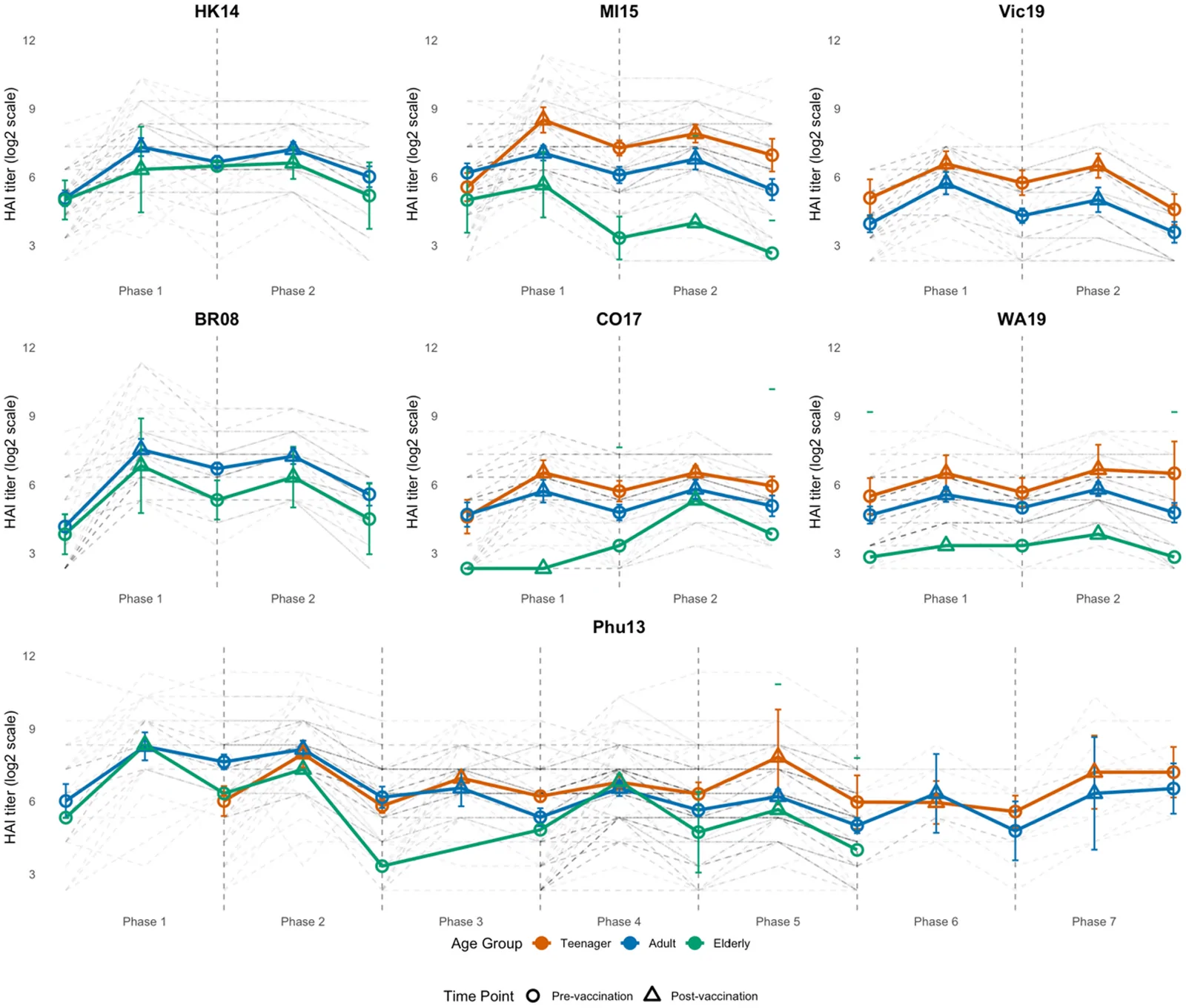
Change of HAI titers among different influenza vaccine strains. Circles represent pre-vaccination (Day 0) HAI titers and triangles represent post-vaccination (Day 21/28) HAI titers. Adjacent phases share the same pre-vaccination Day 0 time point, which is shown once and serves as the endpoint of the preceding waning interval and the starting point of the subsequent vaccination cycle. Each phase includes the vaccine-induced antibody boost from pre-vaccination to post-vaccination and the subsequent inter-season waning from post-vaccination to the next pre-vaccination time point. Vertical dashed lines indicate the boundaries between adjacent phases. Gray dashed lines show individual-level trajectories, colored lines summarize age-group-specific mean trajectories, and vertical error bars indicate 95% confidence intervals.

**Table 1:** Vaccine strains in the UGAFluVac cohort.

Influenza season	Vaccine strains			
	H1N1	H3N2	Yamagata	Victoria
2016/17	CA/09	**HK/14**	**Phu/13**	**BR/08**
2017/18	**MI/15**	**HK/14**	**Phu/13**	**BR/08**
2018/19	**MI/15**	Sing/16	**Phu/13**	**CO/17**
2019/20	BR/18	KS/17	**Phu/13**	**CO/17**
2020/21	GD/19	HK/19	**Phu/13**	**WA/19**
2021/22	**Vic/19**	TAS/20	**Phu/13**	**WA/19**
2022/23	**Vic/19**	DAR/21	**Phu/13**	AS/21

(BR: Brisbane; CA: California; CO: Colorado; GD: Guangdong; HK: Hong Kong; KS: Kansas; MI: Michigan; Phu: Phuket; Sing: Singapore; TAS: Tasmania; Vic: Victoria; WA: Washington; DAR: Darwin; AS: Austria)

**Table 2:** Demographic statistics of participants.

Vaccine strain	First Waning Period	Second Waning Period	Teenager (N, %)	Adult (N, %)	Elderly (N, %)	Gender-Male (N, %)	BMI-median (IQR)	Prior Vaccination-Yes (N, %)
MI/15	2017/18–2018/19	2018/19–2019/20	70, 39.7%	100, 56.8%	6, 3.5%	74, 42.0%	21.73, 30.12	125, 71.0%
Vic/19	2021/22–2022/23	2022/23–2023/24	24, 23.1%	80, 76.9%	0, 0.0%	40, 38.5%	22.96, 31.32	91, 87.5%
HK/14	2016/17–2017/18	2017/18–2018/19	0, 0.0%	115, 89.8%	13, 10.2%	48, 37.5%	22.82, 30.12	176, 100%
BR/08	2016/17–2017/18	2017/18–2018/19	0, 0.0%	96, 88.9%	12, 11.1%	42, 38.9%	23.15, 31.09	75, 69.4%
CO/17	2017/18–2018/19	2018/19–2019/20	52, 39.4%	77, 58.3%	3, 2.3%	56, 42.4%	22.14, 31.80	111, 84.1%
WA/19	2018/19–2019/20	2019/20–2020/21	12, 9.0%	118, 88.1%	4, 2.9%	48, 35.8%	23.81, 33.80	133, 99.3%
Phu/13	–	–	168, 34.0%	319, 64.6%	7, 1.4%	183, 37.0%	22.00, 30.41	329, 66.6%

(For the Phu/13 strain, the first and second dose seasons are not uniform across participants. The numbers reported represent the total participants combined across all available seasons.)

**Table 3:** Coefficients and their 95% CIs for predictors of strain-specific antibody waning (linear mixed effect models).

	H1N1		H3N2		Yamagata		Victoria	
	*n* = 280		*n* = 128		*n* = 494		*n* = 374	
	Coefficients	95%CI	Coefficients	95%CI	Coefficients	95%CI	Coefficients	95%CI
Age Group (Teenager)	0.003	(−0.037, 0.044)	–	–	0.003	(−0.026, 0.030)	−0.006	(−0.053, 0.041)
Age Group (Elder)	0.115*	(0.011, 0.218)	0.025	(−0.030, 0.081)	0.135*	(0.029, 0.240)	0.070*	(−0.064, 0.086)
Gender (Male)	0.005	(−0.017, 0.026)	0.016	(−0.016, 0.050)	0.001	(−0.016, 0.019)	−0.013	(−0.031, 0.006)
BMI	0.002	(−0.000, 0.003)	0.002	(−0.001, 0.005)	0.001	(−0.000, 0.002)	0.001	(−0.000, 0.003)
Prior vaccination (Yes)	0.021	(−0.019, 0.060)	–	–	−0.049***	(−0.077, −0.021)	−0.050•	(−0.105, 0.044)
Pre-vaccination titer	−0.011**	(−0.019, −0.004)	−0.030***	(−0.044, −0.017)	−0.000	(−0.005, 0.005)	−0.000	(−0.007, 0.006)
Boost group (Positive)	0.066***	(0.041, 0.091)	0.048*	(0.006, 0.091)	0.075***	(0.054, 0.096)	0.074***	(0.052, 0.095)
Second Dose	0.011	(−0.017, 0.038)	0.108***	(0.071, 0.147)	0.056***	(0.030, 0.082)	0.049***	(0.028, 0.070)
By strains	Vic/19 VS MI/15	(−0.045, 0.007)	–	–	–	–	BR/08 VS WA/19	(0.048, 0.095)
							0.072***	
	−0.018						CO17/ VS WA/19	(−0.017, 0.029)
							0.006	
Age Group (Teenager) * Second Dose	0.032	(−0.020, 0.085)	–	–	−0.070***	(−0.108, −0.032)	−0.023	(−0.080, 0.034)
Age Group (Elder) * Second Dose	−0.163*	(−0.309, −0.019)	–	–	−0.098	(−0.238, 0.042)	−0.058	(−0.138, 0.023)

*P*-values were derived from Satterthwaite approximation;

‘***’: *p* < 0.001,

‘**’: *p* < 0.01,

‘*’: *p* < 0.05,

‘.’: *p* < 0.1.

In the H3N2 dataset, data for teenagers and individuals without prior year’s vaccination history are not available.

In the H3N2 dataset and the Yamagata dataset, there is only one specific reused strain during our study period.

**Table 4:** Coefficients and their 95% CIs for predictors of strain-specific antibody waning (linear mixed effect models) for the Phu/13 vaccine strain.

	Coefficients	95%CI
Age Group (Teenager)	0.003	(−0.024, 0.029)
Age Group (Elder)	**0.057** •	(−0.024, 0.029)
Gender (Male)	−0.005	(−0.018, 0.007)
BMI	0.001	(−0.000, 0.002)
Prior vaccination (Yes)	−0.053***	(−0.078, −0.029)
**Second Dose**	**0.067** ***	(0.044, 0.088)
Third Dose	−0.016	(−0.041, 0.008)
Fourth Dose	0.011	(−0.017, 0.039)
Fifth or more Dose	−0.005	(−0.037, 0.027)
Pre-vaccination titer	−0.000	(−0.004, 0.003)
Boost group (Positive)	**0.065** ***	(0.050, 0.079)
Age Group* Second Dose (Teenager)	−0.079***	(−0.115, −0.044)
Age Group* Second Dose (Elder)	0.039	(−0.044, 0.121)
Age Group* Third Dose (Teenager)	0.024	(−0.017, 0.066)
Age Group* Third Dose (Elder)	−0.032	(−0.149, 0.085)
Age Group* Fourth Dose (Teenager)	0.015	(−0.039, 0.068)
Age Group* Fourth Dose (Elder)	−0.029	(−0.146, 0.089)
Age Group* Fifth Dose (Teenager)	0.020	(−0.084, 0.125)
Age Group* Fifth Dose (Elder)	−0.078	(−0.216, 0.060)

*P*-values were derived from Satterthwaite approximation;

‘***’: *p* < 0.001,

‘**’: *p* < 0.01,

‘*’: *p* < 0.05,

‘.’: *p* < 0.1.

## Data Availability

Data will be made available on request.

## Appendix A. Supplementary data

Supplementary data to this article can be found online at https://doi.org/10.1016/j.vaccine.2026.129258.
