# Supplementary material for: Faster waning of immunity to influenza vaccine strains reintroduced into subsequent seasons: A human cohort study
Source: Vaccine. Author manuscript; Available in PMC 2026 Nov 7. (PMC13742977; doi:10.1016/j.vaccine.2026.129258)
Supplement: Supplementary file 1 [file NIHMS2219939-supplement-Appendix_A_Supplementary_data.docx]

**Supplementary to “Faster waning of immunity to influenza vaccine strains reintroduced into subsequent seasons: A human cohort study”**

**Supplementary Figure S1. Distributions of pre-vaccination HAI titers across repeated vaccination phases.**

Boxplots show the distributions of pre-vaccination HAI titers for each influenza vaccine strain across successive vaccination phases. The center line represents the median, boxes represent the interquartile range (IQR), whiskers extend to 1.5 times the IQR, and points represent observations beyond the whiskers.

**Sensitivity analysis**

Several sensitivity analyses were conducted to assess the robustness of our findings. First, negative waning scores were set to 0; the statistical significance of the results remained unchanged (**Table S1**) and was consistent with the findings in **Table 3**. Second, individuals without a prior vaccination history were excluded (**Table S2**). Third, we fitted a single mixed-effects model including influenza subtype as a random effect (**Table S3**). Finally, to account for the baseline-dependent ceiling effect, we calculated the pre- to post-vaccination change in HAI titer on the log2 scale and modeled this change as a function of pre-vaccination log2 HAI titer. Individuals with positive residuals were classified as having a positive pre-vaccine adjusted boost and those with non-positive residuals as having a negative pre-vaccine adjusted; this pre-vaccine adjusted boost status was then used in place of the original boost status in the longitudinal mixed-effects model (**Table S4**).

Table S1. Sensitivity analysis in which negative waning scores were set to 0. Coefficients and their 95% CIs for predictors of strain-specific antibody waning (linear mixed effect models). P-values were derived from Satterthwaite approximation; ‘***’: p<0.001, ‘**’: p< 0.01, ‘*’: p< 0.05, ‘.’: p<0.1

|  | | H1N1 | | | H3N2 | | Yamagata | | | Victoria | | |  |
| --- | --- | --- | --- | --- | --- | --- | --- | --- | --- | --- | --- | --- | --- |
|  | | n=280 | | | n=128 | | n=550 | | | n=374 | | |  |
|  | Coefficients | | 95%CI | Coefficients | | 95%CI | | Coefficients | 95%CI | | Coefficients | 95%CI | |
| Age Group (Teenager) | 0.004 | | (-0.037, 0.045) | -- | | -- | | 0.004 | (-0.023, 0.032) | | -0.004 | (-0.036, 0.027) | |
| Age Group (Elder) | **0.121*** | | (0.017, 0.218) | 0.007 | | (-0.042, 0.056) | | **0.143**** | (0.038, 0.250) | | **0.070*** | (0.017, 0.122) | |
| Gender (Male) | 0.001 | | (-0.020, 0.023) | 0.020 | | (-0.012, 0.052) | | 0.005 | (-0.012, 0.022) | | -0.011 | (-0.026, 0.004) | |
| BMI | **0.002*** | | (-0.000, 0.003) | 0.002 | | (-0.001, 0.004) | | 0.001 | (-0.000, 0.002) | | **0.001*** | (0.000, 0.002) | |
| Prior vaccination (Yes) | 0.018 | | (-0.022, 0.058) | -- | | -- | | **-0.043**** | (-0.070, -0.015) | | **-0.044*** | (-0.081, -0.007) | |
| Pre-vaccination titer | **-0.010**** | | (-0.018, -0.003) | **-0.027***** | | (-0.040, -0.014) | | 0.001 | (-0.004, 0.006) | | 0.001 | (-0.004, 0.006) | |
| Boost group (Positive) | **0.073***** | | (0.048, 0.097) | **0.063**** | | (0.025, 0.101) | | **0.077***** | (0.057, 0.097) | | **0.074***** | (0.058, 0.091) | |
| **Second Dose** | 0.013 | | (-0.015, 0.040) | **0.113***** | | (0.077, 0.149) | | **0.052***** | (0.027, 0.077) | | **0.049***** | (0.031, 0.066) | |
| By strains | Vic/19 VS MI/15  -0.013 | | (-0.039, 0.013) | -- | | -- | | -- | -- | | **BR/08 VS WA/19**  **0.064*****  CO17/ VS WA/19  0.002 | (0.045, 0.084)  (-0.016, 0.021) | |
| Age Group (Teenager) * Second Dose | 0.018 | | (-0.033, 0.069) | -- | | -- | | **-0.068**** | (-0.105, -0.032) | | -0.030 | (-0.068, 0.008) | |
| Age Group (Elder) * Second Dose | **-0.164*** | | (-0.309, -0.019) | -- | | -- | | -0.098 | (-0.239, 0.042) | | -0.046 | (-0.115, 0.024) | |

1. In the H3N2 dataset, data for teenagers and individuals without prior year’s vaccination history are not available.

2. In the H3N2 dataset and the Yamagata dataset, there is each only one specific reused strain during our study period.

Table S2. Sensitivity analysis excluding individuals without prior vaccination history. Coefficients and their 95% CIs for predictors of strain-specific antibody waning (linear mixed effect models). P-values were derived from Satterthwaite approximation; ‘***’: p<0.001, ‘**’: p< 0.01, ‘*’: p< 0.05, ‘.’: p<0.1

|  | | H1N1 | | | H3N2 | | Yamagata | | | Victoria | | |  |
| --- | --- | --- | --- | --- | --- | --- | --- | --- | --- | --- | --- | --- | --- |
|  | | n=280 | | | n=128 | | n=550 | | | n=374 | | |  |
|  | Coefficients | | 95%CI | Coefficients | | 95%CI | | Coefficients | 95%CI | | Coefficients | 95%CI | |
| Age Group (Teenager) | -0.061 | | (-0.141, 0.019) | -- | | -- | | -0.047 | (-0.114, 0.022) | | -0.033 | (-0.076, 0.009) | |
| Age Group (Elder) | 0.128* | | (0.027, 0.229) | 0.008 | | (-0.039, 0.057) | | 0.060 | (-0.099, 0.220) | | -0.004 | (-0.058, 0.049) | |
| Gender (Male) | 0.005 | | (-0.019, 0.029) | 0.019 | | (-0.014, 0.053) | | -0.000 | (-0.019, 0.019) | | -0.016 | (-0.032, 0.001) | |
| BMI | 0.002* | | (0.000, 0.004) | 0.000 | | (-0.003, 0.003) | | 0.001* | (0.000, 0.002) | | 0.000 | (-0.001, 0.001) | |
| Pre-vaccination titer | -0.003 | | (-0.012, 0.005) | -0.029*** | | (-0.043, -0.016) | | 0.003 | (-0.003, 0.009) | | -0.003 | (-0.009, 0.003) | |
| Boost group (Positive) | 0.079*** | | (0.054, 0.104) | 0.050* | | (0.014, 0.086) | | 0.064*** | (0.043, 0.085) | | 0.074*** | (0.055, 0.093) | |
| **Second Dose** | 0.011 | | (-0.018, 0.037) | 0.130*** | | (0.093, 0.165) | | 0.056*** | (0.022, 0.067) | | 0.037*** | (0.019, 0.056) | |
| By strains | Vic/19 VS MI/15  0.006 | | (-0.024, 0.035) | -- | | -- | | -- | -- | | BR/08 VS WA/19  0.085***  CO17/ VS WA/19  0.005 | (0.063, 0.107)  (-0.014, 0.024) | |
| Age Group (Teenager) * Second Dose | 0.077 | | (-0.009, 0.163) | -- | | -- | | -0.013 | (-0.084, 0.057) | | 0.006 | (-0.045, 0.056) | |
| Age Group (Elder) * Second Dose | -0.150* | | (-0.291, -0.008) | -- | | -- | | -0.004 | (-0.182, 0.174) | | 0.009 | (-0.061, 0.078) | |

3. In the H3N2 dataset and the Yamagata dataset, there is each only one specific reused strain during our study period.

Table S3. Sensitivity analysis using a single mixed-effects model with subtype included as a random effect. Coefficients and their 95% CIs for predictors of strain-specific antibody waning (linear mixed effect models). P-values were derived from Satterthwaite approximation; ‘***’: p<0.001, ‘**’: p< 0.01, ‘*’: p< 0.05, ‘.’: p<0.1

|  | Coefficients | 95%CI |
| --- | --- | --- |
| Age Group (Teenager) | -0.018 | (-0.042, 0.005) |
| Age Group (Elder) | **0.071**** | (0.029, 0.114) |
| Gender (Male) | -0.000 | (-0.013, 0.013) |
| BMI | **0.001*** | (0.000, 0.002) |
| Prior vaccination (Yes) | **-0.049***** | (-0.070, -0.027) |
| **Second Dose** | **0.050***** | (0.037, 0.063) |
| Pre-vaccination titer | -0.001 | (-0.004, 0.003) |
| Boost group (Positive) | **0.067***** | (0.055, 0.080) |
| Age Group* Second Dose (Teenager) | **-0.028**∙ | (-0.055, 0.000) |
| Age Group* Second Dose (Elder) | -0.017 | (-0.068, 0.033) |

Table S4. Sensitivity analysis using pre-vaccine adjusted boost status. Coefficients and their 95% CIs for predictors of strain-specific antibody waning (linear mixed effect models). P-values were derived from Satterthwaite approximation; ‘***’: p<0.001, ‘**’: p< 0.01, ‘*’: p< 0.05, ‘.’: p<0.1

|  | | H1N1 | | | H3N2 | | Yamagata | | | Victoria | | |  |
| --- | --- | --- | --- | --- | --- | --- | --- | --- | --- | --- | --- | --- | --- |
|  | | n=280 | | | n=128 | | n=494 | | | n=374 | | |  |
|  | Coefficients | | 95%CI | Coefficients | | 95%CI | | Coefficients | 95%CI | | Coefficients | 95%CI | |
| Age Group (Teenager) | 0.005 | | (-0.036, 0.046) | -- | | -- | | 0.004 | (-0.024, 0.032) | | 0.026 | (-0.015, 0.066) | |
| Age Group (Elder) | **0.143**** | | (0.040, 0.248) | 0.028 | | (-0.022, 0.079) | | **0.119*** | (0.015, 0.222) | | **0.070*** | (0.007, 0.132) | |
| Gender (Male) | 0.002 | | (-0.020, 0.023) | 0.012 | | (-0.018, 0.042) | | 0.003 | (-0.014, 0.020) | | -0.016 | (-0.035, 0.003) | |
| BMI | 0.001 | | (-0.000, 0.003) | 0.002 | | (-0.001, 0.004) | | 0.001 | (-0.001, 0.002) | | 0.001 | (-0.001, 0.002) | |
| Prior vaccination (Yes) | 0.026 | | (-0.014, 0.067) | -- | | -- | | **-0.037**** | (-0.065, -0.009) | | 0.013 | (-0.021, 0.046) | |
| Pre-vaccination titer | **-0.018***** | | (-0.026, -0.011) | **-0.044***** | | (-0.055, -0.033) | | **-0.001***** | (-0.014, 0.005) | | **-0.010**** | (-0.016, -0.004) | |
| Pre-vaccination adjusted boost (Positive) | **0.064***** | | (0.040, 0.088) | **0.080***** | | (0.051, 0.109) | | **0.077***** | (0.059, 0.095) | | **0.048***** | (0.028, 0.068) | |
| **Second Dose** | 0.015 | | (-0.013, 0.044) | **0.121***** | | (0.087, 0.155) | | **0.054***** | (0.029, 0.079) | | **0.054***** | (0.031, 0.076) | |
| By strains | Vic/19 VS MI/15  -0.013 | | (-0.039, 0.013) | -- | | -- | | -- | -- | | **BR/08 VS WA/19**  **0.069*****  CO17/ VS WA/19  0.004 | (0.042, 0.096)  (-0.019, 0.027) | |
| Age Group (Teenager) * Second Dose | 0.018 | | (-0.026, 0.079) | -- | | -- | | **-0.068***** | (-0.105, -0.032) | | **-0.066*** | (-0.116, 0.016) | |
| Age Group (Elder) * Second Dose | **-0.180*** | | (-0.325, -0.035) | -- | | -- | | -0.067 | (-0.204, 0.070) | | -0.054 | (-0.138, 0.030) | |

4. In the H3N2 dataset, data for teenagers and individuals without prior year’s vaccination history are not available.

5. In the H3N2 dataset and the Yamagata dataset, there is each only one specific reused strain during our study period.
